# Transsphenoidal Surgery of Giant Pituitary Adenoma: Results and Experience of 239 Cases in A Single Center

**DOI:** 10.3389/fendo.2022.879702

**Published:** 2022-05-06

**Authors:** Yike Chen, Xiaohui Xu, Jing Cao, Yuanqing Jie, Linkai Wang, Feng Cai, Sheng Chen, Wei Yan, Yuan Hong, Jianmin Zhang, Qun Wu

**Affiliations:** ^1^Department of Neurosurgery, The Second Affiliated Hospital, School of Medicine, Zhejiang University, Hangzhou, China; ^2^Department of Neurosurgery, The Fourth Affiliated Hospital, School of Medicine, Zhejiang University, Yiwu, China; ^3^Department of Statistical Office, The Affiliated Changsha Central Hospital, Hengyang Medical School, University of South, Changsha, China; ^4^Department of Neurosurgery, The Affiliated Quzhou People’s Hospital of Wenzhou Medical University, Quzhou, China

**Keywords:** giant pituitary adenoma, transsphenoidal surgery, CSF leak, Knosp grade, extent of resection

## Abstract

**Background:**

Transsphenoidal surgery (TSS) is first-line treatment for giant pituitary adenomas (PAs). Although PA is a benign neuroendocrine tumor that originates from adenohypophysial cells, the surgical outcomes and prognosis of giant PAs differ significantly due to multiple factors such as tumor morphology, invasion site, pathological characteristics and so on. The aim of this study was to evaluate surgical outcomes of giant PAs in a single-center cohort.

**Methods:**

The clinical features and outcomes of 239 patients with giant PA who underwent sphenoidal surgery at the Second Affiliated Hospital of Zhejiang University School of Medicine from January 2015 to October 2021 were collected from medical records. The basic clinical information (age, gender, function etc.), surgical procedure, imaging features (maximum diameter, invasion characteristics, tumor shape etc.) and histopathological characteristics (pathological results, Ki-67, P53 etc.) were retrospectively reviewed. SPSS 25.0 and Stata 12.0 software were used for statistical analysis.

**Results:**

A total of 239 patients with giant PAs underwent TSS, of which 168 surgeries (70.29%) were endoscopic endonasal transsphenoidal (EETS) and 71 (29.71%) were microscopic transsphenoidal (MTS). The mean preoperative maximum diameter in the cohort was 45.64 mm. Gross-total resection was achieved in 46 patients (19.25%), near-total in 56 (23.43%), subtotal in 68 (28.45%), and partial in 69 (28.87%) patients. The maximum tumor diameter and Knosp grade were the significant factors that limited the extent of the resection of giant PAs. A total of 193 patients (80.75%) experienced surgical complications, and the most common complications were postoperative diabetes insipidus (DI) (91, 38.08%), intracranial infection (36, 15.06%) and cerebrospinal fluid (CSF) leaks (37, 15.48%). In addition, there was a significant difference in the incidence of CSF leaks between the neuroendoscopy group and the microscopic group (P < 0.05).

**Conclusion:**

The management of giant PAs remains a therapeutic challenge due to their large size and postoperative complications. The maximum diameter and Knosp grade of giant PAs significantly limited the extent of resection, which warrants a reasonable surgical plan.

## Introduction

Pituitary adenoma (PA) is a benign neuroendocrine tumor that originates from adenohypophysial cells, and accounts for 10%-20% of all primary intracranial tumors (1, 2). Giant PAs are defined as tumors with largest diameter ≥4 cm (3–5), and are characterized by high invasiveness and irregular growth. In addition, giant PAs tend to compresses the optic chiasm and third ventricle, encase the internal carotid artery, and affect hormone secretion from the pituitary gland and hypothalamus (6, 7).

Surgical resection is the first-line treatment for most giant PAs except prolactinoma. Either transcranial or transsphenoidal approaches can be adopted for the surgical removal of giant PAs. Since craniotomy causes greater damage to normal brain tissues and results in more postoperative complications, it is now gradually being replaced with the transnasal sphenoidal approach (8). However, the efficacy of transsphenoidal surgery (TSS) for giant adenomas is poor and is associated with a higher complication rate compared to the smaller PAs (9–11), which can be attributed to the intricate anatomy and secretory functions of the pituitary gland. In this study, we have reviewed the outcomes following TSS of 239 giant PAs from a single-center and analyzed the factors that limit the extent of resection.

## Materials and Methods

### Study Population

The clinical data of 239 patients with giant PAs who underwent TSS at The Second Affiliated Hospital Zhejiang University School of Medicine between January 2015 and October 2021 was retrospectively analyzed. The study was approved by the Research Ethics Committee of SAHZU. The inclusion criteria were as follows: 1) histologically confirmed PAs, 2) maximal diameter of PAs not less than 4 cm according to preoperative MRI, 3) tumor resection through TSS, and 4) regular follow-up for a minimum of 3 months. Patients were excluded if the medical records were not complete, or if the pathological report or follow-up data were missing.

### Data Collection

The basic, surgical, radiological and pathological data was collected. Basic information included age, gender, functional status, clinical presentation. Radiological characteristics included maximum diameter (mm), tumor shape (rounded, dumbbell-shaped, multilobular), invasion characteristics and Knosp classification. Surgical procedures included the surgical method (microscopy or neuroendoscopy), unilateral/bilateral nostrils, the amount of blood loss, the duration of surgery, postoperative hospital stay, extent of resection (gross total resection, GTR (≥95%); near total resection, NTR (≥90%); subtotal resection, STR (≥70%); partial resection, PR [<70%) (12)], endocrine remission and surgical complications. The pathological classification, P53 and Ki-67 positive rates were also collected.

### Tumor Volume Measurement

MRI was typically performed within 2 weeks before surgery and 3 months postoperatively at our institution. The imaging data were obtained through the imaging information-management system. The diameters of the tumors were measured in all direction using a measuring tool in the system, and the extent of resection was determined by comparing pre- and postoperative MRI data.

### Surgical Approach

All patients underwent TSS, and the major surgical procedures were conducted by neurosurgeons with more than 15 years of experience. The objectives of the surgery were to: 1) achieve maximal resection and maximal remission of symptoms with least disturbance to neural and vascular structures, and 2) maintain or reinstate endocrine function.

Patients were positioned supine with the head raised and tilted back slightly. After the induction of general anesthesia, the nasal mucosa and skin of the surgical site was fully disinfected. The following operations are performed under an endoscope (with the aid of a 0° or 30° 4-mm endoscope) or microscope. To avoid nasal mucosal damage, a uninostril approach is used in most cases. Bilateral nostrils technique was performed if the operating space is too narrow. After covering the nasal mucosa with epinephrine cotton pad, the nasal turbinates are lateralized to expand the surgical space. The right pedicled nasoseptal flap was partially resected, then it was stored inferior the surgical channel and was fully harvested if an intraoperative CSF leak occurred. A high-speed drill or osteotome was used to open the sphenoid sinus and the sellar floor was removed. Once the tumor was fully exposed, the lesions localized in the intrasellar and suprasellar region was removed with suction and ring curettes first, then removed the residual lesions in cavernous sinus under direct vision. To protect the carotid arteries and other lateral structures, neuronavigation and Doppler ultrasound were used during resection. Finally, the skull base was reconstructed using the prepared autologous tissue and artificial materials after efficient hemostasis.

### Data Analysis

SPSS 25.0 and STATA 12.0 software were used for statistical analysis. Continuous variables with normal distribution were expressed as mean ± SD and compared by one-way ANOVA. Mann-Whitney U test and Kruskal-Wallis H test were used for categorical variables. Enumeration data were compared using the chi-square and Fisher exact tests. Ordinal logistic regression model was used to identify factors affecting the extent of resection. Two-sided P values < 0.05 were considered statistically significant.

## Results

### General Characteristics

A total of 239 patients (137 females and 102 males) with pathologically confirmed PA were included. The mean age was 51.12 ± 13.8 years (range, 19-84 years). Non-functional PAs was detected in 158 patients (66.11%) and 81 patients (33.89%) had functional PAs. In this series, patients mainly presented with visual impairment 175 (73.22%), including visual acuity (162, 67.78%) and/or visual field (122, 51.05%) deficits. In addition, 67 patients (28.03%) presented with headache, and 41 patients (17.15%) exhibited symptoms of endocrine dysfunction prior to surgery, including irregular menstruation (13, 5.44%), galactorrhea (2, 0.84%), sexual dysfunction (3, 1.26%), acromegaly (6, 2.51%), concentric obesity (2, 0.84%) and thyroid dysfunction (15, 6.28%). Furthermore, 10 patients (4.18%) experienced diabetes insipidus and 23 (9.62%) had apoplexy (confirmed by radiographic and intraoperative findings) prior to surgery (**Table 1**).

**Table 1:** General characteristics.

Variables	Value*
Age (years)	
mean ± SD	51.12 ± 13.80
median	53
range	19-84
Gender	
male	102 (42.68)
female	137 (57.32)
Functional Status	
nonfunctioning	158 (66.11)
functioning	81 (33.89)
Clinical presentation	
headache	67 (28.03)
visual acuity deficits	162 (67.78)
visual field deficits	122 (51.05)
irregular menstruation	13 (5.44)
galactorrhea	2 (0.84)
sexual dysfunction	3 (1.26)
acromegaly	6 (2.51)
concentric obesity	2 (0.84)
thyroid dysfunction	15 (6.28)
diabetes insipidus	10 (4.18)
apoplexy	23 (9.62)

*Values are number of patients (%) unless stated otherwise.

The average maximum diameter for the giant PAs was 45.64 ± 6.7 mm (range 40–75 mm). The tumors were round in 14 cases (5.86%), dumbbell shaped in 89 cases (37.24%), and multilobular in 136 cases (56.9%). Based on the preoperative MRI results and intraoperative observations, 220 cases (92.05%) had cavernous sinus invasion, 165 (69.04%) had sphenoid sinus invasion and 221 cases (92.47%) showed suprasellar invasion. The highest Knosp grade was 0-1 for 26 patients (10.88%), 2 for 46 patients (19.25%), 3A for 38 patients (15.90%), 3B for 21 patients (8.79%) and 4 for 108 patients (45.19%), which were indicative of the extent of cavernous sinus invasion and aggressiveness of the giant PAs (**Table 2**).

**Table 2:** Radiological characteristics.

Variables	Value*
Maximum Diameter (mm)	
mean ± SD	45.64 ± 6.70
median	44
range	40-75
Tumor Shape	
rounded	14 (5.86)
dumbbell shaped	89 (37.24)
multilobular	136 (56.90)
Invasion Characteristics	
cavernous sinus invasion	220 (92.05)
sphenoid sinus invasion	165 (69.04)
suprasellar invasion	221 (92.47)
Knosp Grade	
0-1	26 (10.88)
2	46 (19.25)
3A	38 (15.90)
3B	21 (8.79)
4	108 (45.19)

*Values are number of patients (%) unless stated otherwise.

As expected, the predominant pathological type of the giant PAs was gonadotrophic adenomas (76, 31.8%), followed by lactotroph adenomas (27, 11.3%), corticotroph adenomas (27, 11.3%), pluri-hormonal and double adenomas (22, 9.21%), null cell adenomas (20, 8.4%), and somatotroph adenomas (3, 1.26%). 64 pathological results that were not based on latest WHO criteria and lack of transcription factors evaluation were excluded. Furthermore, Ki-67 labeling index was ≥ 5% in 19 patients (7.95%), < 3% in 178 patients (74.48%) and 3%-5% in 42 patients (17.57%). Positive staining for P53 was 14.64%, and 200 patients (83.68%) were negative and 4 patients (1.67%) had weak staining (**Table 3**).

**Table 3:** Pathological characteristics.

Variables	Value*
Cell Type	
Somatotroph adenomas	3 (1.26)
lactotroph adenomas	27 (11.30)
TSH adenomas	0 (0.00)
corticotroph adenomas	27 (11.30)
gonadotrophic adenomas	76 (31.80)
null cell adenomas	20 (8.4)
Pluri-hormonal and double adenomas	22 (9.21)
unknown	64 (26.78)
Ki-67	
<3%	178 (74.48)
3%-5%	42 (17.57)
≥5%	19 (7.95)
P53	
negative	200 (83.68)
positive	35 (14.64)
weak	4 (1.67)

*Values are number of patients (%) unless stated otherwise.

### Surgical Procedure

All patients underwent TSS, of which 168 patients (70.29%) were treated with neuroendoscopy and 71 (29.71%) with microscopy. The average operating duration was 143.18 ± 80.26 minutes (range, 46-475 minutes) and the mean intraoperative blood loss was 160.46 ± 285.71ml (range, 10-3500 ml). Four cases had more than 1000 ml of intraoperative blood loss. In addition, the two-nostril approach was taken in 39 cases and 200 patients were treated with the one-nostril approach. The mean length of stay after surgery was 9.59 ± 7.52 days and 7 patients were hospitalized for more than 1 month, mainly because of endocrine dysfunction and intracranial infection. According to postoperative MRI, GTR was achieved in 46 cases (19.25%), NTR in 56 cases (23.43%), STR in 68 cases (28.45%) and PR in 69 cases (28.87%). Improvement of vision was achieved in 133 patients (76.00%). Endocrine tests were performed 3 days, 1 week, and 3 months postoperatively. Sixty-five patients with functional giant PAs achieved endocrine remission after TSS. All details are summarized in **Table 4**.

**Table 4:** Surgical characteristics.

Variables	Value*
Surgical Method	
neuroendoscopy	168 (70.29)
Microscopy	71 (29.71)
Unilateral/Bilateral Nostrils	
unilateral nostrils	200 (83.68)
bilateral nostrils	39 (16.32)
Blood Loss	
mean ± SD	160.46 ± 285.712
median	100
range	10-3500
Operating Duration (min)	
mean ± SD	143.18 ± 80.26
median	125
range	46-475
Postoperative Length of stay (days)	
mean ± SD	9.59 ± 7.52
median	8
range	1-49
Extent of Resection	
PR	69 (28.87)
STR	68 (28.45)
NTR	56 (23.43)
GTR	46 (19.25)
Visual Improvement	133 (76.00)
Endocrine Dysfunction	
non-remission	10 (4.18)
remission	65 (27.20)

GTR, gross total resection; NTR, near total resection; STR, subtotal resection; PR, partial resection. *Values are number of patients (%) unless stated otherwise.

A total of 193 patients (80.75%) experienced surgical complications (**Table 5**), and the most common complication was postoperative diabetes insipidus (DI), which occurred in 91 patients (38.08%). The incidence of intracranial infection and cerebrospinal fluid (CSF) leaks was 15.48% (13) and 15.06% (14) respectively. Furthermore, 18 patients with CSF leaks developed secondary intracranial infections, and 8 patients experienced postoperative intracranial hemorrhage due to abundant tumor blood supply and inadequate hemostasis of residual tumor. Cranial nerve palsies were observed in 5 cases, all of whom experienced impaired visual field and limited eye movement. One patient experienced intraoperative internal carotid artery injury and was discharged after interventional therapy. Two patients died of intracranial infection and multiorgan failure. In addition, there was a significant difference in the incidence of CSF leaks between the neuroendoscopy and the microscopy groups (P < 0.05) (**Table 6**).

**Table 5:** Surgical complications.

Variables	No. of Patients (%)
CSF leaks	36 (15.06)
DI	91 (38.08)
Intracranial infection	37 (15.48)
Epistaxis	0 (0.00)
Intracranial hemorrhage	8 (3.35)
Cranial nerve palsies	5 (2.09)
Internal carotid artery injury	1 (0.42)
Death	2 (0.84)

CSF leaks, cerebrospinal fluid leaks; DI, diabetes insipidus.

**Table 6:** Complications comparison of ETTS and MTTS.

Surgical Complications	EETS (n = 168)	MTS (n = 71)	X^2^	P
CSF leaks	32 (19.05)	4 (5.63)	7.019	0.008
DI	64 (38.1)	27 (38.03)	0.000	1.000
Intracranial infection	28 (16.67)	9 (12.68)	0.607	0.436
Epistaxis	0 (0.00)	0 (0.00)	0.000	1.000
Intracranial hemorrhage	3 (1.79)	5 (7.04)	–	0.053^#^
Cranial nerve palsies	4 (2.38)	1 (1.41)	–	1.000^#^
Internal carotid artery injury	1 (0.60)	0 (0.00)	–	1.000^#^

EETS, endoscopic endonasal transsphenoidal; MTS, microscopic transsphenoidal; CSF leaks, cerebrospinal fluid leaks; DI, diabetes insipidus. ^#^means using Fisher’s exact test.

### Risk Factors of Extent of Resection

The effect of various tumor characteristics on the extent of resection are outlined in **Table 7**, factors (P > 0.05) including age, gender, functional status, surgical method, unilateral/bilateral nostrils, tumor shape, invasion characteristics, Ki-67 labeling index, P53 were not significantly correlated with the extent of resection. Univariate analysis showed the maximum diameter of giant PAs maybe a significant factor limiting the extent of resection (P < 0.05). In the ordinal logistic regression model, the OR of maximum diameter was 0.95 (P < 0.05; 95%CI: 0.92-0.98) (**Table 8**). The Knosp grade was showed a significant effect on the extent of resection (P < 0.001). GTR was more likely achieved in giant PAs with lower Knosp grade, especially Knosp grade 0-1 (P < 0.05; OR: 2.96; 95%CI: 1.27, 6.90) (**Table 8**).

**Table 7:** The effect of various tumor characteristics on the extent of resection.

Variables	PR (n = 69)	STR (n = 68)	NTR (n = 56)	GTR (n = 46)	x^2^/F	*P*
Gender					0.682	0.878
M	32 (46.38)	29 (42.65)	23 (41.07)	18 (39.13)		
F	37 (53.62)	39 (57.35)	33 (58.93)	28 (60.87)		
Age (years)	51.64 ± 14.95	51.59 ± 12.83	53.00 ± 11.42	47.35 ± 15.66	1.562	0.199
Functional Status					6.920	0.075
N	47 (68.11)	42 (61.76)	32 (57.14)	37 (80.43)		
Y	22 (31.89)	26 (38.24)	24 (42.86)	9 (19.57)		
Surgical Method					6.921	0.074
neuroendoscopy	42 (60.87)	52 (76.47)	37 (66.07)	37 (80.43)		
microscopy	27 (39.13)	16 (23.53)	19 (33.93)	9 (19.57)		
Unilateral/Bilateral Nostrils						
unilateral nostrils	60 (86.96)	57 (83.82)	44 (78.57)	39 (84.78)	1.655	0.647
bilateral nostrils	9 (13.04)	11 (16.18)	12 (21.43)	7 (15.22)		
Maximum Diameter (mm)	48.26 ± 8.14	44.72 ± 6.37	44.8 ± 5.46	44.09 ± 5.05	5.333	0.001
Tumor Shape					_	0.095^#^
rounded	3 (4.35)	2 (2.94)	2 (3.57)	7 (15.22)		
dumbbell shaped	21 (30.43)	29 (42.65)	25 (44.64)	14 (30.43)		
multilobular	45 (65.22)	37 (54.41)	29 (51.79)	25 (54.35)		
Cavernous Sinus Invasion					_	0.065^#^
N	3 (4.35)	10 (14.71)	5 (8.93)	1 (2.17)		
Y	66 (95.65)	58 (85.29)	51 (91.07)	45 (97.83)		
Sphenoid Sinus Invasion					1.577	0.665^#^
N	24 (34.78)	21 (30.88)	18 (32.14)	11 (23.91)		
Y	45 (65.22)	47 (69.12)	38 (67.86)	35 (76.09)		
Suprasellar Invasion					–	0.259^#^
N	2 (2.9)	7 (10.29)	6 (10.71)	3 (6.52)		
Y	67 (97.1)	61 (89.71)	50 (89.29)	43 (93.48)		
Knosp Grade					18.417	<0.001
0-1	8	4	3	11		
2	12	11	8	15		
3	13	16	21	9		
4	36	37	24	11		
Ki-67					0.781	0.854
<3%	49	51	42	36		
3%-5%	15	10	9	8		
≥5%	5	7	5	2		
P53					1.819	0.611
negative	60	54	46	40		
positive	7	13	9	6		
weak	2	1	1	0		

GTR, gross total resection; NTR, near total resection; STR, subtotal resection; PR, partial resection. ^#^means using Fisher’s exact test.

**Table 8:** Ordinal logistic regression for factors of GTR.

Variables	P	OR	95%CI
Knosp Grade			
0-1	0.012	2.96	1.27, 6.90
2	0.009	2.52	1.26, 5.07
3	0.031	2.03	1.07, 3.88
4	–	1.00	–
Maximum Diameter	0.004	0.95	0.92, 0.98

## Discussion

Although relatively rare, giant PAs present significant challenges in terms of surgical resection and postoperative management on account of their size and frequent invasion into the surrounding normal tissues. In this study, we retrospectively analyzed the clinical data and surgical outcomes of 239 patients with giant PAs, and identified risk factors for the extent of resection.

Around 6-10% of PAs are defined as giant PAs based on their largest diameter (15, 16). A total of 2829 patients with PAs were treated at our center from January 2015 to October 2021, of which 8.4% had giant PAs. The frequency of the clinically non-functioning giant PAs (158; 66.11%) was twice as high as that of the functioning adenomas (81; 33.89%). This finding is consistent with that reported by Pedro et al. (6). This could be due to the difficulty in detecting silent PAs till they grow to a certain size and become symptomatic. Furthermore, 16 cases of clinically non-functioning giant PAs were confirmed as corticotroph adenomas, which are commonly found in large size of PAs and have been recognized as a more aggressive subtype of pituitary adenomas (17).

TSS is the first-line treatment for giant PAs (18) except the prolactinomas that can be effectively treated with dopamine agonists. The main goals of surgical resection of PAs are the restoration of normal pituitary function, nerve and vascular decompression, and minimal damage to the surrounding tissues. Since the 1990s, the endoscopic endonasal transsphenoidal (EETS) approach has been widely practiced for its improved surgical visualization (19–22), since endoscopes with angled lenses can be used to access areas that are not visible under a microscope. Komotar et al. conducted a systematic review (1995–2010) to compare the outcomes of EETS and microscopic transsphenoidal approach (MTS), and found the EETS group had higher rates of GTR (47.2%) compared to the MTS group (30.9%) (23). Michael et al. further reported significantly higher mean reduction of tumor volume with EETS (91%) compared to MTS (63%) in a cohort of 72 patients with giant PAs (21). In this study, the extent of resection in the EETS group was higher than that in the MTS group, albeit without statistical significance.

Giant PAs are associated with a higher surgical complication rate compared to normal PAs, and the most common complications are DI, CSF leaks, postoperative intracranial hemorrhage, intracranial infections, cranial nerve palsies, hypopituitarism and epistaxis (12, 24, 25). Consistent with previous reports (25–28), the three most frequent complications in our cohort were DI (91, 38.08%) and 6 patients developed permanent DI, CSF leaks (36, 15.06%), and intracranial infection (37, 15.48%). DI is caused by posterior pituitary disfunction, and its incidence rate typically ranges from 9% to 22%, and may increase up to 53% at some centers (29–34). Nevertheless, only 2-7% of the patients develop permanent DI (35,36). PA patients with visual abnormalities, suprasellar extension or large tumors are at a higher risk of developing DI postoperatively (36), which could be the reason for the high incidence of DI in our cohort.

CSF leaks are generally the result of surgical injury and tumor invasion, especially in case of giant PAs with anterior cranial fossa extension (37) and suprasellar expansion (14). In our cohort, most cases of CSF leaks occurred during tumor removal. After reconstructing the skull base with the vascularized nasoseptal flap or the fascia and subcutaneous fat of the thigh, the CSF leaks in most patients receded within 3-6 days. In addition, we found that the rate of CSF leaks was higher in the EETS group than in the MTS group, which was consistent with the results of Yoshua et al. (13). This may be related to the imaging features of neuroendoscopy, which only provides 2D images without the important depth and three-dimensional sense. A previous study reported a significant association between CSF leak and postoperative intracranial infection (38), indicating that despite advanced skull base reconstruction and antibiotic treatment, some patients with CSF leaks may still develop intracranial infection. In this study, 37 patients experienced intracranial infection and 2 patients died as a result. Therefore, intracranial infection still represents a common and feared complication of this approach.

In our cohort, postoperative intracranial hemorrhage was reported in 8 (3.35%) cases. This is a terrible postoperative complication, which can cause great risk to the patient’s life and economic pressure. Residual tumor and inadequate hemostasis were main reasons for this complication, so extreme caution should be exercised after giant PAs surgery. Once a hematoma in the operative cavity is found, an early evacuation of the hematoma and decompression of cranial nerve are urgent needed.

Internal carotid artery rupture is a rare complication but carries the greatest risk of short-term mortality, and there are reports describing fatal bleeds from damaged carotid arteries (39–41). In this study, one patient experienced internal carotid artery damage and formed a false aneurysm, which was managed by interventional endovascular treatment. In addition, cranial nerves are easily damaged during resection of tumors invading the cavernous sinus. Therefore, maintaining a strictly midline approach, familiarity with MRI results and the use of Doppler ultrasound is essential for neurovascular protection.

Since gross total resection is the optimal surgical outcome of giant PAs, we identified independent risk factors of the extent of resection in order to plan a suitable surgical strategy. Tumor size and the invasiveness of giant PAs into surrounding structures are key factors that limit the extent of resection. In our study, we found that each 1 mm increase in tumor diameter corresponded to a 5% decrease in the chance of achieving a GTR. Likewise, giant PAs with Knosp grades 0-1, 2 and 3 were more than twice as likely to achieve GTR compared to those with grade 4 (**Table 8**). Therefore, both the maximum diameter and the Knosp grade are independent factors of the extent of resection. Sanmillan et al. (42) also identified tumor volume and the Knosp grade as independent risk factors of the extent of resection in a study conducted on 294 patients with PAs, and found that the Knosp grade had a greater impact. Consistent with this, we found that some giant PAs with low Knosp grade could be satisfactorily removed despite their large size. Thus, cavernous sinus invasion of the PAs is crucial for planning surgical procedures, and tumor size can provide complementary information.

The primary aim of identifying risk factors limiting the extent of resection is to guide the neurosurgeons to distinguish the operation terminal and avoid complex complications rather than achieve complete removal of giant PAs. Therefore, the ultimate goals of the surgical resection of giant PAs are decompression of neurovascular structures, especially the optic nerve and internal carotid artery, relieving endocrine dysfunction and controlling the tumor progression, instead of gross total resection of the giant PAs. However, this study has certain limitations that are largely related to its single-center retrospective nature, and a longer follow-up and multicenter cohort are needed to validate our results.

## Data Availability Statement

The raw data supporting the conclusions of this article will be made available by the authors, without undue reservation.

## Ethics Statement

The studies involving human participants were reviewed and approved by Ethics Committee of Second Affiliated Hospital, School of Medicine, Zhejiang University. Written informed consent for participation was not required for this study in accordance with the national legislation and the institutional requirements.

## Author Contributions

YC and XX performed the analysis and co-wrote the manuscript. LW, XX, YJ, FC, SC, and JC collected the patient information. WY and YH revised paper. QW and JZ supervised the project, conceived the study, and guided the editing of the manuscript. YC and XX contributed equally to the manuscript. QW and JZ are corresponding authors. All authors contributed to the article and approved the submitted version.

## Conflict of Interest

The authors declare that the research was conducted in the absence of any commercial or financial relationships that could be construed as a potential conflict of interest.

## Publisher’s Note

All claims expressed in this article are solely those of the authors and do not necessarily represent those of their affiliated organizations, or those of the publisher, the editors and the reviewers. Any product that may be evaluated in this article, or claim that may be made by its manufacturer, is not guaranteed or endorsed by the publisher.
